# Move to Improve: Co‐Designing a Hospital‐Based Physical Activity Program for Children With Chronic Health Conditions

**DOI:** 10.1111/hex.70628

**Published:** 2026-03-01

**Authors:** Hamsini Sivaramakrishnan, Amy Finlay Jones, Treya Long, Louise Naylor, Jane Valentine, Lisa Martin, Louise Haustead, Vinutha Shetty, Fiona Wood, Elizabeth Davis, Move to Improve Author Group

**Affiliations:** ^1^ The Kids Research Institute Australia Nedlands Australia; ^2^ School of Medicine University of Western Australia Crawley Australia; ^3^ School of Population Health Curtin University Bentley Australia; ^4^ Child and Adolescent Mental Health Service Nedlands Australia; ^5^ School of Human Sciences University of Western Australia Crawley Australia; ^6^ Perth Children's Hospital, Child and Adolescent Health Service Nedlands Australia; ^7^ School of Biomedical Sciences University of Western Australia Crawley Australia; ^8^ Fiona Wood Foundation Murdoch Australia; ^9^ Burns Service of Western Australia Murdoch Australia

**Keywords:** adolescent, chronic disease, chronic illness, exercise, sport, young people

## Abstract

**Background:**

Physical activity can support physical and mental health among children living with chronic health conditions; however, programmes must be tailored to their specific needs to support participation.

**Objective:**

This study aimed to co‐design the protocol for Move to Improve, a hospital‐based clinical service at Perth Children's Hospital to support physical activity participation among children with chronic health conditions.

**Methods:**

Four online co‐design workshops were conducted with children living with chronic health conditions and their parents, using a participatory and collaborative approach to inform programme development. Data were analysed using reflexive thematic analysis.

**Results:**

Five interrelated themes were identified, highlighting the importance of family‐led and goal‐oriented approaches, individualised programme design, enjoyment, confidence building and support for transition to community‐based physical activity. Together, these findings underscore the need for family‐centred and tailored programmes that support sustained engagement in physical activity.

**Conclusion:**

The co‐design process informed the development of a structured protocol incorporating evidence‐based strategies aligned with families' priorities. These strategies included goal setting at the start of the programme, goal review at the end of the programme and tailoring of strategies such as skill building and education on self‐management of the condition across the duration of the programme. This protocol offers a clinically relevant and scalable model for supporting physical activity participation among children with chronic health conditions within hospital‐based services.

**Patient and Public Contribution:**

Children and parents of children living with chronic health conditions participated in co‐design workshops, and their input directly informed and shaped key elements of the Move to Improve protocol, including programme structure, content and delivery strategies.

## 1 Introduction

Chronic health conditions that require ongoing medical attention are common, with about 13%–27% of children affected worldwide [1]. These conditions can significantly impact the health and well‐being of children and their families [2]. For instance, children with chronic health conditions such as Type 1 diabetes and asthma can experience poorer quality of life, and are thrice as likely to experience emotional, developmental and behavioural difficulties when compared with their peers without such conditions [3, 4]. Furthermore, high rates of co‐occurring mental health conditions have been noted in these children [3]. Therefore, there is a need to develop interventions that can reduce such difficulties and support positive outcomes for children with chronic health conditions.

Physical activity is well‐regarded as a primary prevention tool for several chronic health conditions [5]. More recently, the utility of physical activity in the management and prevention of complications associated with chronic health conditions is also gaining widespread recognition [6, 7]. A breadth of evidence indicates that physical activity may be associated with physical and psychosocial benefits for children living with a wide range of chronic health conditions [6, 8, 9, 10]. These benefits include increased strength and cardiorespiratory fitness, reduced blood pressure and depressive symptoms and improved quality of life [7, 11, 12]. Physical activity can also help manage condition‐related symptoms (e.g., managing blood glucose levels in Type 1 diabetes, reducing fatigue following cancer treatment) and prevent complications associated with the condition [7].

While numerous policies and guidelines recommend regular physical activity for healthy Australian children and adolescents, there are few that have been developed with consideration of the needs of young people with chronic health conditions [13, 14]. Targeting physical activity in childhood is particularly important, as being physically active in childhood increases the likelihood of being a healthy and active adult [15]. As outlined above, children with chronic health conditions have specific requirements that need to be considered to support their participation in physical activity and overcome challenges associated with access, inclusion and safe participation. Involvement of young people with chronic conditions in the development of such programmes would ensure that the needs of these individuals are considered when developing guidelines and programmes to support their engagement in physical activity [16]. Co‐design is a methodology that involves collaboration between researchers and consumers (i.e., the target group for an intervention, policy or other output that is being designed) to develop solutions that meet consumers' needs [17]. Co‐design has been successfully utilised to develop interventions to improve the health and well‐being of children and young people living with chronic health conditions [18, 19].

### 1.1 The Present Study

This study is part of Move to Improve, an ongoing programme of research that aims to co‐design and implement a clinical service at Perth Children's Hospital to support children with chronic health conditions to increase their participation in physical activity. Building on a systematic review [20] and a series of semi‐structured interviews, the present study aimed to work collaboratively with children living with chronic health conditions and their parents/primary caregivers to co‐design the protocol for the Move to Improve service and associated pilot trial. Specifically, we aimed to (1) identify key touch points across the duration of the programme, and (2) ascertain strategies that could be embedded at each of these touch points to encourage participation in Move to Improve and the maintenance of physical activity participation into the future.

## 2 Methods

### 2.1 Context

The Move to Improve service will be hospital‐based and is designed to support children with chronic health conditions to increase their participation in physical activity in a manner that supports health and well‐being outcomes. The service will be implemented and evaluated at Perth Children's Hospital. Move to Improve will initially be piloted with four chronic health conditions (i.e., Type 1 diabetes, burn injuries, cerebral palsy and cancer survivors). Evidence gathered from this pilot study will inform the expansion of the service to other chronic health conditions and support plans to embed the service as part of standard care. The protocol for the Move to Improve pilot study has been developed using a co‐design research framework informed by multiple sources of evidence (i.e., Expert Reference Group, interviews with families, literature review). The findings of the interviews (under review) and literature review [20] have been reported elsewhere. This paper reports on the co‐design of Move to Improve and how these multiple sources of evidence informed the design and development of the programme protocol.

### 2.2 Informing the Co‐Design Process

An Expert Reference Group comprising clinicians (e.g., medical doctors, allied health staff) and researchers was engaged at the beginning of the programme of research and met monthly to navigate and inform key decisions. Given their familiarity with the hospital system and in working with children with chronic health conditions, this group informed key decisions pertaining to the feasibility and acceptability of delivering the proposed programme. When developing the discussion guides and materials, we consulted with the Expert Reference Group to identify questions that clinicians had for families in terms of how they would like the Move to Improve programme to be delivered. These questions were subsequently incorporated into activities that were developed for the co‐design workshops.

**Figure 1:**
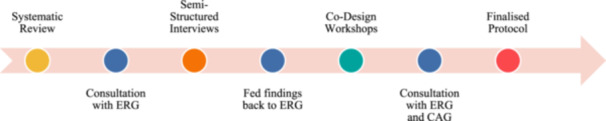
Process map outlining the co‐design process.

In addition to input from clinical experts, before commencement of the co‐design workshops, we conducted a systematic review to identify behaviour change techniques commonly used within physical activity interventions for children with chronic health conditions [20]. The review identified 35 promising behaviour change techniques that could support short‐ and long‐term participation in physical activity and positive psychosocial outcomes. We also conducted a series of interviews with children who had received treatment at Perth Children's Hospital for their chronic health conditions and their parents/primary caregivers. The purpose of these interviews was to generate a broad understanding of barriers and facilitators for engaging in physical activity and to identify what families would want to see in a hospital‐based physical activity programme. The findings of these interviews informed the design of the discussion guides and workshop materials. For instance, we constructed case studies that embedded the barriers and facilitators described by families in the interviews. These case studies were discussed during the co‐design workshops to ensure the provided examples were realistic and relatable, and helped identify solutions to these challenges. The co‐design process map is illustrated in Figure 1.

### 2.3 Co‐Design Workshops

Ethical approval for this study was obtained from the Government of Western Australia Child and Adolescent Health Service Human Research Ethics Committee (approval number: RGS5994). A series of co‐design workshops was held in March and April 2024 to inform the development of Move to Improve. These workshops involved an experience‐based co‐design (EBCD) approach. EBCD is a participatory research approach that requires collaboration between key stakeholders and focuses on designing user experiences and identifying key touch points where improvement may be necessary. EBCD has been used in a variety of healthcare settings, including primary care and mental health services, for quality improvement purposes [21]. This approach utilises broad brainstorming processes and narratives such as stories and scenarios to help communicate ideas and how these might be utilised in action. This process helps with identifying potential pitfalls and solutions that meet the needs of all stakeholders involved.

Four, 2 h co‐design workshops were planned to be conducted on a weekly basis to enable scaffolding of the discussion across the weeks. These workshops were ultimately offered at two different times in the week to accommodate the availability of different families. Convenience sampling was used to identify eight families (parent and child) who were available and willing to participate in a series of four workshops spanning 8 h of discussion. As the pilot study for Move to Improve focuses on children aged 5–18 years living with either Type 1 diabetes, burn injuries, cerebral palsy or cancer, we focused on these children (and their parents) in our co‐design process. The demographic details of children and parents attending the co‐design workshops are presented in Table 1.

**Table 1:** Demographic details of co‐design workshop participants.

Identifier	Gender	Condition	Age
Parent 1	Female	Type 1 diabetes (son)	Not collected
Parent 2	Female	Type 1 diabetes (son)	Not collected
Parent 3	Female	Type 1 diabetes (son)	Not collected
Parent 4	Female	Cerebral palsy (son)	Not collected
Parent 5	Female	Cerebral palsy (daughter)	Not collected
Parent 6	Female	Burn injury (daughter)	Not collected
Parent 7	Female	Burn injury (daughter)	Not collected
Parent 8	Female	Cancer (son)	Not collected
Child 1	Male	Type 1 diabetes	8
Child 2	Male	Type 1 diabetes	10
Child 3	Male	Type 1 diabetes	9
Child 4	Male	Cerebral palsy	15
Child 5	Female	Cerebral palsy	10
Child 6	Female	Burn injury	15
Child 7	Female	Burn injury	9

All parents were given information sheets before the first workshop and were asked to provide informed consent for their own participation and on behalf of their child. Children were asked to provide verbal assent to participate. Parents and children were given a gift card for each workshop attended in appreciation for their time. All families attended all four workshops, with four families attending each available timeslot. Having smaller groups and maintaining continuity each week allowed for genuine rapport to be built between families, which in turn fostered rich discussions at each workshop. All workshops were held in English and conducted online using Microsoft Teams based on families' unanimous preference for the accessibility afforded by this format. The workshops were facilitated by two members of the Move to Improve team, one researcher and one programme manager. All workshops were recorded and transcribed verbatim. All workshop materials were disseminated online for discussion, and the electronic artefacts were stored digitally.

### 2.4 Discussion Guides and Workshop Activities

Based on the input received from the Expert Reference Group and families interviewed, we designed discussion guides for four co‐design workshops. The discussion guides were designed to be a series where each workshop would build on the discussion of the previous one. Broadly, the focus of each workshop was:

- Workshop 1: What would help children engage in physical activity and participate in Move to Improve?
- Workshop 2: What supports and strategies could be embedded within Move to Improve to encourage participation?
- Workshop 3: What supports and strategies could help families complete the research assessments?
- Workshop 4: Reflections and final feedback on the programme and research plans.
- Four case studies (one representing each of the chronic health conditions of interest) were purposively constructed in collaboration with clinicians to be realistic representations of a patient in the hospital receiving treatment for a specific condition. These case studies incorporated the barriers and facilitators outlined in the interviews we conducted, as well as the literature more broadly. These case studies were used in activities during the workshops, allowing families to draw on relevant experience to generate ideas without needing to reveal their personal circumstances in a group setting.

Workshop activities included brainstorming, where families were asked to respond to a series of prompts and generate ideas using online interactive polls via Mentimeter. Their responses were visible in real time in the form of word clouds. Storyboards were also used to map out participants' journey in the programme, and families were encouraged to identify strategies that may be relevant to incorporate across this timeline to help support their participation. Families were also provided with some materials (i.e., assessment protocol and questionnaires) to review in their own time and provide written feedback via email. Group discussions were facilitated using open‐ended questions across all workshops to gather detailed input from families on all aspects of programme design and implementation. The discussion guides are presented in Table 2.

**Table 2:** Workshop discussion guides.

Workshop theme	Activities
Workshop 1: What would help children engage in physical activity and participate in Move to Improve?	Group discussion: How do we talk about physical activity to children and young people living with chronic health conditions? Word cloud: What physical activity goals would children and parents want help with? Case studies: ◦ What would encourage families to get involved with Move to Improve? ◦ What barriers would they experience to participation? ◦ What would be important to help them sustain participation in Move to Improve?
Workshop 2: What supports and strategies could be embedded within Move to Improve to encourage participation?	Group discussion: What does the journey of a Move to Improve participant look like? Brainstorm: What supports and strategies would be important to incorporate within Move to Improve to encourage continued participation? Sticky notes: At what stage(s) of the journey should specific strategies be embedded?
Workshop 3: What supports and strategies could help families complete the research assessments?	Review research assessments to be completed: ◦ Physical testing ◦ Questionnaires Individual feedback: ◦ How acceptable are the proposed research assessments at baseline and follow‐up timepoints? ◦ What supports and strategies would help with completing assessments?
Workshop 4: Reflections and final feedback on the programme and research plans	Reflect on discussion from workshops 1–3 Reflect on revised protocol incorporating feedback from workshops 1–3

### 2.5 Data Analysis

The data from the co‐design workshops were analysed iteratively. A preliminary analysis was conducted at the end of each week to summarise key ideas raised in the session. These findings informed discussion prompts for the following week's workshops. This method allowed for continuity in discussion by scaffolding information from one workshop to the next. Given the participatory nature of the co‐design approach, facilitators made notes following each session, and the preliminary themes were discussed with the Expert Reference Group at the halfway point. These notes and discussions helped shape the discussion guides for subsequent workshops. Once all the workshops were completed, transcripts, notes and artefacts were collated and analysed using reflexive thematic analysis [22] to identify key themes to inform the development of Move to Improve. Using the six‐phase process, the lead author engaged in a process of data familiarisation and initial coding, followed by clustering of initial codes. Codes were revisited to identify themes, which were then reviewed with a co‐author to consolidate key themes. These themes were then shared with families to allow them to provide further comment, which allowed for further refining of themes. The themes were also discussed with the Expert Reference Group in order to determine how to feasibly implement the themes in practice. Together, the recommendations and feedback were incorporated to finalise the protocol for the Move to Improve pilot study.

## 3 Results

Building on the themes identified during the interviews with families, across the co‐design workshops, we identified five main themes capturing families' views on the implementation and delivery of Move to Improve. Specifically, families highlighted ways in which Move to Improve could be designed and delivered to accommodate families' preferences while attempting to engage them in a hospital‐based physical activity programme: ‘A family‐led, goal‐oriented approach’, ‘An individualised programme addressing family concerns’, ‘Putting fun at the forefront’, ‘Increasing confidence to be active’ and ‘Supporting transition to community‐based physical activity’.

### 3.1 A Family‐Led, Goal‐Oriented and Community‐Focused Approach

Families expressed that a physical activity programme such as Move to Improve would benefit from being guided by children's and their families' physical activity goals. This approach was considered important to fostering children's and families' motivation to participate in Move to Improve. Collaboratively setting goals with clinicians would allow families to feel a sense of ownership in physical activity participation. Developing an intervention tailored to each child, and aligned with their intrinsic motivation, was considered crucial to sustained engagement in Move to Improve and achieving goals could motivate children towards long‐term physical activity more generally.

I like the idea of setting goals … to try to tap into any competitive impulses that kids may or may not have. I know some kids are just completely turned off by the prospect of being sort of measured and set challenges, but then with [my child], I know that that's an effective thing.

– Parent of a child living with Type 1 diabetes

Families also favoured a community‐focused approach that could help them build connections locally, thereby making physical activity participation easier and more enjoyable. A community‐focused approach would also ensure that physical activity participation could be sustained following Move to Improve.

It's all about if the family and the child can carry on, then improvement will continue and that's when you will gain the most out of the programme. If you can facilitate and set this child up to continue to exercise, it doesn't matter if through dance, through the beach, through community soccer, through community swimming—it doesn't matter what it is—if the child will continue to participate on the regular basis…that's where the most out of this exercise will come.

– Parent of a child living with cerebral palsy

I think they need to be part of something and feel like they're a part of it, and that they fit somewhere in this community.

– Parent of a child living with Type 1 diabetes

### 3.2 An Individualised Programme to Suit Different Families

For Move to Improve to be of practical relevance to families in the long run, it was considered important to adopt an individualised approach that was responsive to the child's specific medical condition and families' circumstances. This would allow clinicians to tailor the programme to each family's needs, address specific challenges and minimise barriers to physical activity.

What works for one family doesn't work for another.

– Parent of a child living with Type 1 diabetes

I'm a single parent. I work, you know, we don't have a lot of time to go and do stuff. My kids are tired after school. Like, I want them to be active, but sometimes the logistics are the biggest factor in not allowing things to happen.

– Parent of a child living with burn injuries

There is a lack of established physical activity protocols for children with different chronic health conditions. Hence, of specific importance is the necessity to identify means of engaging in physical activity while adhering to condition‐specific management protocols and following advice from medical practitioners. Move to Improve, as an embedded hospital‐based service, could support this need by liaising with a child's multidisciplinary medical team to ensure their advice is considered when supporting physical activity, while also feeding relevant physical activity information back to the medical team for further consideration in relation to the child's ongoing course of treatment.

These kids [with acute lymphoblastic leukemia] have a pump [medical device]…So it's about introducing something that allows the child to move without interfering with the safety protocols that are applied to cancer patients at times, especially kids.

– Parent of a child who was diagnosed with cancer

### 3.3 Putting Fun at the Forefront

Children prioritised friendships, team‐based activities and sports when thinking about physical activity settings. Children expressed a desire for a physical activity programme that could help them make friends, demonstrating the value of connections built through participating in physical activity.

Through my netball club I met a lot of good friends, [who] now are some of my best friends at school.

– Teenager living with burn injuries

I feel good when I do [team sport] … like, just being with other people and actually having some competition … I feel like I belong.

– Child living with Type 1 diabetes

The most common outcomes parents wanted for their child when participating in Move to Improve were for them to be able to have fun and connect with others. They viewed such connections as integral to fostering positive experiences in physical activity environments. Fun and connection were considered important factors in motivating children to sustain physical activity participation.

I think fun is an important word…we know that they need the health and that is vital, but they don't necessarily want that, especially if you've got kids dealing with so much other crap in their life…you want them to have fun.

– Parent of a child who was diagnosed with cancer

### 3.4 Increasing Confidence to Be Active

Given the need for an individualised programme to support children with chronic health conditions to participate in physical activity, an initial one‐to‐one approach that could help children build their confidence to engage in physical activity was important. This would support initiation and maintenance of physical activity participation throughout and after Move to Improve. This is something families felt that Move to Improve should strive to achieve in the early weeks of the programme. Families suggested two ways to build children's confidence: by supporting skill development specific to physical activity and through cultivating peer connections for an ongoing sense of community.

When kids at a very young age are dealing with a chronic condition, obviously it does have an impact on their emotional wellbeing, and also their sense of autonomy…an exercise programme can make him feel more empowered.

– Parent of a child living with Type 1 diabetes

I think even building their capacity to talk about their modifications or whatever they need if they're going to engage with their friends and their peers in an effective way would be good too.

– Parent of a child living with cerebral palsy

The need for education on self‐management of the condition was also discussed as a way to help boost parents' confidence in caring for their child living with a chronic health condition, and specifically to support their participation in physical activity.

When he is able to get to a point of being more confident and we feel confident in his self‐management, then obviously, it enables us to look after ourselves [as parents] a little bit better.

– Parent of a child living with Type 1 diabetes

Subsequently, once initial confidence was addressed, families wanted the latter part of the programme to focus on integration into community physical activity settings (described further below). Families also articulated a need to be involved in regular discussions surrounding physical activity with their treating medical team, especially following Move to Improve, to ensure long‐term safety and confidence in engaging in physical activity.

With us, it's still an ongoing challenge really, to plan for [my child's] blood glucose control and management when he is physically active, because there's so many different factors involved. We have a generic plan, which is a component within his endocrinology‐endorsed action plan…but one thing for us that would be really useful is if there was a cross‐relationship between your programme and then like perhaps the CDEs [clinical diabetes educators] or the endocrinologists…so that, you know, there's actual practical outcomes and suggestions about how families can better manage different levels of exercise…Those would be useful selling points.

– Parent of a child living with Type 1 diabetes

### 3.5 Supporting Transition to Community‐Based Physical Activity

In participating in a physical activity programme, families emphasised the importance of feeling a sense of community and the role of this in supporting initial and long‐term engagement. This sense of community was important for children due to the motivation that being with other children of a similar age or ability could provide. Families suggested that a key aim of Move to Improve should be to link children and families with clubs, groups and supports within their own communities. One way in which Move to Improve could support families would be to help them identify community‐based programmes that are suitable for their child, and help them build up the skills and confidence required to partake in that programme.

What I think the programme should be is about that connection, and giving the kids a sense of belonging, community.

– Parent of a child living with cerebral palsy

While Move to Improve as a programme would need to be individualised, families did seem to value some group‐based elements in having opportunities for socialising (particularly with those of a similar age or ability, or similar physical activity goals) and working together toward goals, while emphasising the importance of having fun.

We were talking about that…about how powerful connection with people walking the same or similar path is…Where opportunities exist for some kind of group activities or group connections, that would be really good.

– Parent of a child living with Type 1 diabetes

I really would be keen to see in programmes chances for the kids to connect with their peers, but also the kids to connect with older kids, for that mentoring aspect.

– Parent of a child living with cerebral palsy

Equally, for parents, meeting other parents of children of a similar age or ability would allow them to connect and learn from each other's experiences. This was something families welcomed and hoped would be incorporated into Move to Improve to allow for long‐term support channels once the programme had ended.

Connecting with other parents that are doing the programme, sometimes we can problem solve with each other.

– Parent of a child living with cerebral palsy

Linking the family to a community group is also support and encouragement to carry on.

– Parent of a child living with cerebral palsy

### 3.6 Mapping of Design Elements

The themes identified from the co‐design workshops and perspectives of the Expert Reference Group were reviewed to identify design elements that could be incorporated within the protocol for the Move to Improve pilot study. To support this process, we mapped promising behaviour change techniques identified in our review of physical activity interventions for children with chronic health conditions (see [20]) to the Theoretical Domains Framework [23], using the Theory and Techniques tool to identify potential mechanisms of action to support short‐term and long‐term engagement in physical activity. These multiple sources of information were synthesised to narrow down key elements relevant to different stages across the participant journey. For instance, the list of strategies discussed by families during the co‐design workshops was reviewed alongside the most promising behaviour change techniques in the systematic review to narrow down a shortlist of key strategies. This process ensured that the protocol incorporated families' perspectives while remaining rooted in evidence‐based practice. Specific elements were planned to be introduced at the start of the programme, the end of the programme, as well as across the duration of the programme as required (see Table 3).

**Table 3:** Mapping of systematic review evidence and co‐design workshop themes to strategies embedded within programme protocol.

Timepoint	Mechanisms of action^a^	Co‐design workshop themes	Strategies embedded within protocol
Start of programme	Goals	A family‐led, goal‐oriented approach	Goal setting Action planning (programme‐oriented) Identifying sources of social support
During programme	Beliefs about capabilities Skills	An individualised programme addressing family concerns Putting fun at the forefront Increasing confidence to be active	Confidence building Education on self‐management of condition Skill development Integrating into community settings
End of programme	Behavioural regulation	Supporting transition to community‐based physical activity	Goal review Action planning (future‐oriented)

^a^ Obtained from the Theories and Techniques Tool when inputting promising behaviour change techniques associated with short‐term and long‐term physical activity behaviour (from Sivaramakrishnan et al. [20] systematic review).

At the start of the programme, families deemed it important to set achievable goals to work towards throughout the programme. This was further endorsed when mapping behaviour change techniques, with ‘Goals’ identified as a promising mechanism of action to help initiate short‐term physical activity [20]. Importantly, families also reinforced the importance of having goals that are led by the child and their family to work towards, and highlighted the positive implications these can have for physical activity participation. Hence, in the Move to Improve programme, goal‐setting will involve exploring a child's physical activity interests, identifying barriers to activity engagement and working collaboratively with a member of the Move to Improve team delivering the programme to identify a few physical activity goals to work towards over the course of the programme. A previous hospital‐based intervention designed to promote physical activity behaviour change for children with cancer also identified collaborative goal setting with a healthcare professional as important in developing a tailored programme [24, 25]. In that programme, goal setting helped children have something to work towards and facilitated regular review [24]. This approach also aligned with the international clinical practice guideline for children with cerebral palsy, which recommends identifying and working towards child‐led goals, and highlights the role of clinicians in supporting identification of specific barriers that may limit participation [14].

Building a sense of connection was also identified as key in supporting children's participation in physical activity. This included connecting the child and their family with appropriate physical activity providers and identifying sources of social support (e.g., participating with a family member, friends) to encourage the child to work towards their physical activity goals [26]. noted the importance of supportive peer relations in physical activity settings for children with chronic conditions. Parents also play a pivotal role in helping manage their child's chronic health condition while participating in physical activity [26]. Such forms of social support have effectively been mobilised in other interventions for this target group [27, 28].

Across the 8‐week Move to Improve programme, several elements were identified as being potentially relevant to incorporate. When mapping behaviour change techniques, ‘beliefs about capabilities’ and ‘skills’ were identified as promising mechanisms of action relevant to initiation and maintenance of physical activity in the long run [20]. Across interviews and co‐design workshops, families highlighted that confidence building, education on self‐management of the condition while engaging in physical activity, skill building and integrating activity into community settings would support engagement in physical activity. Noting the importance of tailored advice and physical activity programmes, we aim to design Move to Improve to be tailored to each child's physical activity goals and decided that any specific areas of concern would be identified upon commencement of the programme. Tailoring has been noted as pivotal to effectively engaging individuals with chronic conditions in a wide range of self‐management interventions, including physical activity [29]. A tailored approach ensures that interventions are specifically adapted to suit the individual child and the wide range of social and environmental barriers that may inhibit engagement with an intervention. Tailoring interventions would also allow for consideration of the child's developmental stage and degree of dependency on parents/primary caregivers [30].

In the Move to Improve programme, the need for confidence building will be addressed by helping children address barriers towards physical activity participation and working towards small goals to allow a sense of accomplishment. This strategy has been effectively utilised in other interventions to encourage progression towards physical activity goals and has also been recommended in clinical practice guidelines [14, 24]. Education will involve providing families with further understanding of managing a chronic health condition while participating in physical activity. Such approaches have been widely utilised in healthcare settings, with demonstrated effectiveness in managing chronic health conditions in children and increasing physical activity levels [29]. Additionally, such education has been shown to be effective in improving parents' quality of life [31].

Regarding the need for skill building, Move to Improve will involve isolating specific physical skills required to achieve physical activity goals and working towards developing these skills through tailored programming. Skill development has been found to be especially important in fostering physical activity self‐efficacy; in children with chronic health conditions, a strong association was noted between sport participation and self‐efficacy for exercise [7]. Integrating activity into community settings will involve identifying and working toward transitioning into appropriate and suitable community‐based activity options to encourage sustained participation. As specified in the international clinical practice guideline for children with cerebral palsy, children should work towards physical activity goals within their home or community settings to increase the likelihood of these activities being incorporated into everyday life once interventional support has ended [14].

At the end of the programme, families identified that incorporating goal review and action planning was especially important for physical activity maintenance once support from the Move to Improve programme had ended. When mapping behaviour change techniques, ‘behavioural regulation’ was identified as a promising mechanism of action to help sustain long‐term participation in physical activity [20]. Therefore, in line with existing evidence [14, 24], goal review will be embedded to support children to experience a sense of achievement, build confidence and sustain motivation for physical activity. Constructing an action plan that will enable the child to become more independent when participating in community‐based physical activity was also considered an important component of the Move to Improve programme. Action planning has been noted as an important strategy to incorporate within tailored interventions to develop an individualised physical activity plan to empower the child to continue to engage in physical activity in the long run [32]. It is hoped that incorporating these elements into the programme will support self‐management and sustained engagement in physical activity once the Move to Improve programme has been completed.

## 4 Discussion

The aim of this study was to co‐design Move to Improve, a hospital‐based behaviour change programme to support children with chronic health conditions to increase their participation in physical activity and support health and well‐being outcomes. The co‐design of clinical programmes to support physical activity behaviour change with children living with chronic health conditions, their families and clinicians is limited [33]. used a co‐design approach to develop prototypes of interventions to support children with physical disabilities to increase their engagement in physical activity. Working with parents, clinicians and other key stakeholders, intervention prototypes focused on behaviour change techniques that were considered important to encourage physical activity participation (e.g., building self‐efficacy, autonomy and setting goals) were developed. Following a theoretical design process [25], the study utilised the Behaviour Change Wheel to inform the development of a hospital‐based physical activity intervention for children undergoing acute cancer treatment. The resulting 10‐week programme, CanMOVE, was considered feasible to deliver within a hospital setting. The implementation of strategies such as individualised goal setting, professional support and monitoring was considered especially important to supporting behaviour change [24].

The protocol for the Move to Improve programme was developed with extensive input from multiple sources, including co‐design workshops, Expert Reference Group meetings and research evidence. In doing so, it is hoped that key barriers to implementation and uptake of Move to Improve have been considered at the outset, thereby encouraging feasibility and acceptability of the programme. The planned implementation of the Move to Improve programme is as follows: (1) initial intake appointment, (2) 8‐week programme, (3) post‐programme assessment. Children referred into the programme will undergo an initial intake appointment at Perth Children's Hospital—this session will involve a collaborative goal‐setting exercise by a member of the Move to Improve team with the child and their family to determine individualised physical activity goals to work towards throughout the programme. Additionally, children will also be asked to complete some physical tests to understand baseline physical capacity and guide the development of an individualised programme to help them work towards their set physical activity goals. Children will then take part in an 8‐week behaviour change programme to support sustained engagement in physical activity. Over the 8‐week programme, participants will have access to 1 h/week one‐to‐one physical activity sessions with a member of the Move to Improve team (e.g., exercise physiologist, physiotherapist). These sessions will focus on addressing barriers to achieving the identified physical activity goals by applying a combination of strategies, including confidence building, physical skill development, education on self‐management of conditions and support with integration into community‐based physical activity programmes. This approach will allow the strategies applied at each session to be individualised to each child's physical activity goals and the barriers and facilitators relevant to their circumstances. Where psychology or dietetic input is identified as relevant to goal achievement, these services will be provided on a case‐by‐case basis in addition to the weekly physical activity sessions. Following completion of the 8‐week programme, children and their families will partake in a goal review exercise with a member of the Move to Improve team to reassess progress towards their physical activity goals. Families will also be supported to identify appropriate community‐based physical activities to encourage continued participation once the programme has ended.

## 5 Strengths and Limitations

A key strength in our work was the use of an EBCD approach, which permitted key stakeholders to identify key touch points and co‐design the experience of the Move to Improve programme. By incorporating a variety of activities and discussions with a diverse group of families, we were able to capture a wide range of perspectives to co‐design a model of care that is transdiagnostic and, therefore, potentially easier to scale up when translating the research into practice. The structure of the co‐design workshops (i.e., conducting a series of four workshops, once a week for 4 weeks) permitted continuity, but also allowed families to build rapport with each other and with the facilitators. This ensured that open and rich discussions were possible, allowing us to capture genuine perspectives and useful information to guide the design and implementation of the programme. We also incorporated evidence‐based findings to support and validate our programme design to ensure that while the programme was co‐designed, it was also evidence‐based in its practice.

There are some limitations to consider with this work. It is important to note that the families, at times, provided suggestions that we were unable to feasibly incorporate into the protocol. For instance, families emphasised the importance of connection and role modelling and suggested matching each child with an appropriate member of the Move to Improve team (e.g., a male physiotherapist to work with teenage boys) to support their physical activity progression. However, in practice, matching may be challenging to achieve; staff capacity would likely dictate allocation of participants to specific team members. It is also worth noting that all the parents involved in the workshops were mothers. While these mothers identified as the child's primary caregiver, there remains a gap in capturing the perspectives of fathers who may also be primary caregivers of children living with chronic health conditions. While we included families representing perspectives of four different chronic health conditions in the co‐design workshops, a limitation arises in that we did not include other stakeholder groups (e.g., healthcare professionals, hospital executives) in these workshops. While this meant that stakeholder perspectives were not captured within the co‐design workshops, we intentionally chose to exclude other stakeholders from this process to allow families to freely express their opinions without concern for how expressing their views might impact their child's medical care. Instead, we incorporated stakeholder perspectives by involving the Expert Reference Group at key decision timepoints, keeping them up to date with families' perspectives and seeking their professional input. This approach meant that families' perspectives were considered to ensure acceptability of the programme, while the clinical perspectives ensured feasibility of programme delivery in a hospital setting.

## 6 Conclusion

This paper has detailed the collaborative development of Move to Improve through a co‐design approach that involved children living with chronic health conditions and their parents, regular input from key stakeholders and a review of the literature. These multiple sources of information have allowed for the varied priorities of families and healthcare professionals to be simultaneously considered to ensure that Move to Improve is tailored to the needs of these different stakeholders. The result is a programme protocol that is acceptable and engaging for families that is also feasible for clinicians to deliver within a hospital setting. This protocol will be utilised as part of a pragmatic trial of the Move to Improve programme to assess the effectiveness and scalability of the programme in supporting sustained physical activity participation in children with chronic health conditions in a manner that supports health and well‐being outcomes.

## Author Contributions

**Hamsini Sivaramakrishnan:** conceptualisation, methodology, investigation, formal analysis, project administration, writing – original draft. **Amy Finlay‐Jones:** conceptualisation, methodology, supervision, funding acquisition, writing – review and editing. **Elizabeth Davis:** conceptualisation, funding acquisition, writing – review and editing. **Louise Naylor:** funding acquisition, writing – review and editing. **Jane Valentine:** funding acquisition, writing – review and editing. **Fiona Wood:** funding acquisition, writing – review and editing. **Treya Long**, **Lisa Martin**, **Louise Haustead** and **Vinutha Shetty:** writing – review and editing. Move to Improve Author Group: conceptualization, funding acquisition.

## Ethics Statement

The study has received ethics approval from the Government of Western Australia Child and Adolescent Health Service Human Research Ethics Committee (RGS5994).

## Conflicts of Interest

The authors declare no conflicts of interest.

## Data Availability

The original full‐length transcripts will not be fully released to protect participant privacy in line with the study's ethics protocol.
